# Evaluation of flaxseed lignan-enriched extract targeting autophagy, apoptosis, and hedgehog pathways against experimentally induced obesity

**DOI:** 10.5455/javar.2023.j684

**Published:** 2023-06-30

**Authors:** Safaa I. Khater, Maram Shalabi, Buthainah B. Alammash, Alaa I. Alrais, Doaa S. Al-Ahmadi, Leena S. Alqahtani, Tarek Khameis, Sahar Abdelaziz, Amr Elkelish, Kh. El-Dawy

**Affiliations:** 1 Department of Biochemistry, Faculty of Veterinary Medicine, Zagazig University, Zagazig, Egypt; 2 King Fahad Hospital, Ministry of Health, Medina, Saudi Arabia; 3 Maternity and Children Hospital (MCH), Ministry of Health, Medina, Saudi Arabia; 4 Department of Biochemistry, College of Science, University of Jeddah, Jeddah, Saudi Arabia; 5 Department of Pharmacology, Laboratory of Biotechnology, Faculty of Veterinary Medicine, Zagazig University, Zagazig, Egypt; 6 Department of Pharmacognosy, Faculty of Pharmacy, Zagazig University, Zagazig, Egypt; 7 Department of Biology, College of Science, Imam Mohammad Ibn Saud Islamic University (IMSIU), Riyadh, Saudi Arabia

**Keywords:** Autophagy, antioxidant, apoptosis, flaxseed extract, lignans, obesity, phytoestrogens

## Abstract

**Objective:**

This research investigated secoisolariciresinol diglucoside (SDG) flax extract effects on apoptosis, hedgehog (Hh), autophagy, and the anti-oxidation process in experimentally induced obesity.

**Materials and Methods:**

Forty rats were separated into two sets regarding either receiving a normal balanced diet or a high-fat diet (HFD) and then distributed into four groups: GI: The control group had a regular diet for 12 weeks. GII: animals received a high-fat meal and saline by gastric gavage. GIII: HFD obese rats treated with SDG extract orally (10 mg/kg/b.w.) and 1.18 mg SDG/kg in the diet for 4 weeks GIV: Normal balanced diet rats received SDG extract orally (10 mg/kg/b.w.) and 1.18 mg SDG/kg of chow for 12 weeks in addition to their regular balanced diet.

**Results:**

The administration of SDG extract exhibited a significant drop in body weight, glucose, lipid profile, and leptin compared to the obese group. It also improved the antioxidant levels (lowering the levels of malondialdehyde while increasing the total antioxidant capacity) and anti-inflammatory status (decreasing interleukin-6 and tumor necrosis factor-alpha). SDG extract downregulates the expression of HH genes (protein patched homolog 1, Hh-interacting protein, glioma-associated oncogene homolog 1, and smoothened receptor) in conjunction with the modulation of autophagy genes and apoptotic proteins.

**Conclusion:**

SDG extract showed improved anti-inflammatory and antioxidant status and downregulated the expression of HH genes while modulating autophagy genes and apoptotic proteins among obese rats, suggesting that it may be used to avert and manage obesity and its correlated complications by modulating oxidation, inflammation, autophagy, and apoptosis. Advanced future research on the SDG autophagy pathway to address obesity and its complications is mandatory.

## Introduction

Obesity has reached epidemic proportions, affecting approximately 1.9 billion people globally and causing 2.8 million deaths a year [1]. Despite the potential to prevent and treat obesity by understanding the ongoing advances in its pathophysiology, obesity treatments are still limited [2]. Women going through menopause may experience an increased visceral adipose tissue volume and an increased risk of metabolic problems, which may be mitigated to some extent by hormone replacement treatment [3]; accordingly, estrogen-like chemicals may be used to combat obesity.

Phytoestrogens are dietary substances obtained from plants that are chemically similar to human estrogens and have biological functions. As a result, they were first labeled as substances that affect the endocrine system [endocrine-disrupting chemicals (EDCs)]. Phytoestrogens are classified as selective estrogen modulators or regulators based on their principal mechanism of action [4]. Plant-based estrogens may provide various health benefits [5,6]. There is some inconsistency in the impact of phytoestrogens on the composition of the body and obesity induction, which suggests that phytoestrogens’ effect on adipose tissue distribution and metabolism is complicated and affected by additional variables [7].

Secoisolariciresinol diglucoside (SDG) is a core lignan in linseed. It also includes additional phytochemical nutrients regarded as essential prospective anti-inflammatory substances since the standard anti-inflammatory medications now in use address acute inflammatory disorders but are ineffective against chronic inflammatory ailments. Natural phytochemical antioxidants are safe replacements for synthetic antioxidants with significant adverse effects [8,9]. In addition to its nutritional advantages, this seed is also regarded as a functional food because of its positive effects on metabolism and physiology. As there is a significant tendency to use auxiliary conventional medical care, the number of people using it has steadily risen [10]. As a supplement, several studies have shown it to be beneficial in improving the lipid profile, reducing the progression of diabetes mellitus (types I and II), controlling weight gain and obesity, and reducing blood pressure [10,11].

Phytoestrogens have pro-adipogenic, antioxidant, apoptotic, anti-mutagenic, and anti-angiogenic properties and can modify transcriptional factors and microRNA. The pro-adipogenic pathway of phytoestrogens in the modulation of obesity is related to a reduction of insulin (INS) resistance, activation of estrogen receptors (ERs), inhibition of reactive oxygen species, activation of antioxidants, and decreases of lipoprotein lipase, fatty acid synthase, and cyclic adenosine monophosphate [12,13]. Also, phytoestrogens’ anti-inflammatory and apoptotic effects through blocking NF-KB and tumor necrosis factor (TNF) in obese rats enhanced apoptosis, decreased the interleukins associated with pro-inflammation, and decreased the secretion of leptin [14,15].

Recent research has shown a unique method of phytoestrogen activity involving autophagy control. Autophagy is a fundamental cellular procedure that eradicates inactive organelles and proteins. Phytoestrogens could either stimulate or obstruct autophagy initiation, depending on whether autophagy activation would cause cell death or survival [16–19]. Obesity and diabetes mellitus were prevented and treated using autophagy regulators since these statistics propose that autophagy deficiency resulted in the conversion of obesity to diabetes [20].

Specific inflammatory cytokines are produced in response to increased fat stores during obesity [interleukin-6 (IL-6) and TNF]. There are two ways in which inflammation might affect autophagy. Adipose tissue autophagy flux is augmented by obesity-induced inflammatory cytokines, particularly TNF-α, via upregulating autophagic genes [21]. Hence, the mammalian target of rapamycin (mTOR) is regarded as a link between inflammation and autophagy [22,23]. Autophagy markers were higher in the adipose tissue of obese mice in contrast to those of lean controls, as reported by Nuñez et al. [24]. This process might be reversed by dietary changes.

Hedgehog (Hh) signaling is a preserved natural progression that regulates tissue growth and function. Adipocyte development is suppressed *in vitro* as Hh signaling is activated [25,26]. Due to its apparent contribution to adipogenesis, the Hh signaling process has been recommended as a possible therapeutic approach for metabolic conditions, including type II diabetes and obesity [27,28].

Even though many plants are currently employed in food consumption and medication manufacturing, herbal medicines seem to be an underused weapon for preventing many diseases [29–31]. These data make studying novel natural effects derived from plants an immediate concern. SDG has been used in inhibiting and managing some chronic diseases; however, scarce data are found regarding autophagy and Hedgehog pathways in the modulation of obesity. Accordingly, the present study aimed at understanding the mode of action of the SDG flax extract as a nutraceutical supplement in the modulation of obesity through different mechanisms, including apoptosis, the *h*-Hog pathway, autophagy, and anti-oxidation.

## Materials and Methods

### Ethical approval

The research was permitted by the Zagazig University Research Center Institutional Animal Care and Use Committee (IACUC) (Approval No. ZU-IACUC/2/F/208/2022).

### Animal protocol

After receiving ethical approval and consent, the animal experiment was done at the Animal House of the Veterinary Medicine Faculty at Zagazig University, Egypt. The institution provided 40 male Wister rats with a weight ranging from 175 to 280 gm kept in plastic polycarbonate cages with 12-h dark/light cycles and unrestricted admission to diet and drink. Forty rats were randomized into two main groups for the investigation (20 rats in each group) according to receiving either a normal diet or a high-fat diet (HFD). The rats were kept in the animal house for 2 weeks to acclimate to their new environment. Rats were given pelleted commercial food and water *ad libitum*. The traditional food and the HFD were prepared depending on the study of Lasker et al. [32]. The SDG, ≥97% [high-performance liquid chromatography (HPLC)] enriched lignan, was purchased from Sigma Aldrich, Product Number: S0202, CAS Number: 148244-82-0. It is listed among the natural products at the company. SDG is a pure lignan extracted from flaxseed through HPLC with ≥97% purity.

SDG in the diet was prepared according to a previous study with some modifications [33] at a dose of 1.18 mg/kg of diet. The oral dose (10 mg/kg of body weight) was dissolved in water and given to the animals daily using gastric gavage [34].

### Experimental design

The rats were separated into four groups (Table 1). GI: The control group was nourished with a standard regime for 12 weeks. GII: an HFD for 8 weeks supplemented with saline via a gastric gavage tube with a normal diet for 4 weeks. GIII: 10 rats were fed with HFD for 8 weeks, after which they were administered SDG flaxseed extract orally (10 mg/kg/b.w.) and 1.18 mg SDG/kg in the diet for the remaining 4 weeks [33]. GIV: control normal diet group that received SDG flaxseed extract orally (10 mg/kg/b.w.) and 1.18 mg/kg of food for the next 12 weeks.

### Sampling

#### Blood samples

After 12 weeks, the groups were sedated with 30 mg/kg IP of 2.5% thiopental sodium, and the cardiac blood was collected by cardiac puncture and separated into two sections. The initial blood samples were obtained in empty tubes for serum isolation and held at −20°C until the laboratory processes were completed. The anesthetized rats were sacrificed by head displacement for dissection. The remaining half was collected into screw-capped tubes with sodium fluoride as an anticoagulant and centrifuged at 3,000 rpm for 15 min to isolate the plasma, which was used directly for blood glucose and other measurements.

**Table 1.** Type of diet and daily dose intake for each group.

Treatments		
GI	Normal diet	Regular diet for 12 weeks
GII	Obese	HFD
GIII	Obese + SDG	HFD (8 weeks) + SDG extract orally (10mg/kg/b.w) and 1.18 mg SDG/kg/diet (4 weeks)
GIV	Normal control diet +SDG	Standard diet (8 weeks) + SDG extract orally (10 mg/kg/b.w) and 1.18 mg SDG/kg/diet (4 weeks)

#### Tissue samples

Liver and adipose tissue samples were subdivided into three after scarifying. The first part was enfolded with aluminum foil and placed instantly in liquid nitrogen to snap-freeze tissue and decrease endogenous RNase for real-time polymerase chain reaction (RT-PCR) gene expression investigation. The second part was covered with foil and stored at −20°C until malondialdehyde (MDA) and total antioxidant capacity (TAC) determination. The third component was kept in 10% buffered formalin for histopathology and immunohistochemistry.

### Body weight

All animal groups were weighed prior to experimentation, after 8 weeks (initial body weight), and at the termination of the research to determine the final weight.

### Biochemical tests

Blood glucose estimation (BioMed diagnostic kit, Egypt), serum lipid profile (Randox assay kits), serum aspartate aminotransferase (AST/GOT, CAT: MAK080/MAK006) activity [35], MDA (Rat MDA ELISA Kit Cat No. MBS268427), and (TAC Assay Kit Cat No. MAK187) were conducted by using colorimetric assays following the manufacturer specifications. The instructions were followed to quantify serum TNF-α and IL-6 levels using ELISA (Thermofisher Scientific CO., Cat: KRC3012, Cat: ERA31RB, respectively). The determination of serum INS concentration was done by the RayBio^®^ rat INS enzyme-linked immunosorbent assay (ELISA kit method). Leptin level measurements were done using the R&D Systems ELISA kit (catalog number MOB00; R&D Systems). Plasma levels of adiponectin were evaluated using an ELISA kit (Millipore, USA).

### Quantitative polymerase chain reaction

Total RNA was extracted from adipose tissue using the TRIzolTM reagent (Invitrogen, ThermoFisher Scientific, Waltham, MA) (Catalog Numbers 15596026 and 15596018). The HiSenScriptTM RH (-) cDNA Synthesis Kit (iNtRON Biotechnology Co., South Korea) was used for cDNA synthesis. Sangon Biotech has created primers suited for the desired genes (Beijing, China) (Table 2). The RT-PCR was conducted using an Mx3005P Real-Time PCR System (Agilent Stratagene, USA) and TOPrealTM qPCR 2X PreMIX (SYBR Green with low ROX) provided by applied enzynomics at Life Technologies (India) Pvt. Ltd. The target gene expression levels were adjusted using the mRNA expression of a known housekeeping gene, Gapdh. Using the 2-CT approach, results are reported as fold changes relative to the control group [36].

### Histopathologic examinations

Specimens of liver and perirenal fat were fixed, dehydrated, cleaned in xylene, implanted in paraffin, and sectioned at a width of 5 m. A part of the paraffin slices was marked with hematoxylin and eosin (H&E) [37], inspected, and photographed using a light microscope.

**Table 2.** Primers used in determination of gene expression of the selected genes.

Gene	Forward primer (5′–3′)	Reverse primer (5′–3′)	Accession No
Gapdh	GCATCTTCTTGTGCAGTGCC	TACGGCCAAATCCGTTCACA	NM_017008.4
Beclin-1	GAATGGAGGGGTCTAAGGCG	CTTCCTCCTGGCTCTCTCT	NM_001034117.1
LC-3	GAAATGGTCACCCCACGAGT	ACACAGTTTTCCCATGCCCA	NM_012823.2
mTOR	GCAATGGGCACGAGTTTGTT	AGTGTGTTCACCAGGCCAAA	NM_019906.2
P62	GGAAGCTGAAACATGGGCAC	CCAAGGGTCCACCTGAACAA	NM_181550.2
SMO	TTCCTCATCCGAGGGGTCAT	ATTGATCTTGCTGGCTGCCT	NM_012807.1
Gli-1	CCTCCACCCCAGTATCTCCA	ACAATTCCTGCTGCGACTGA	NM_001191910.1
Ptch-1	TCCCCTCCTCCTCCTCTTTC	CTTGTTCTCCTCACCGACCC	NM_053566.3
Hhip	GCTCTTTGGTCCTGATGGCT	GCTGGTTGGTGCTGTTGAAG	NM_001191817.1
AMPK	GTACCCTGGCAGCAGGATTT	TCCAGGATGGCAACGAAGTC	NM_022627.2

**Figure 1.**
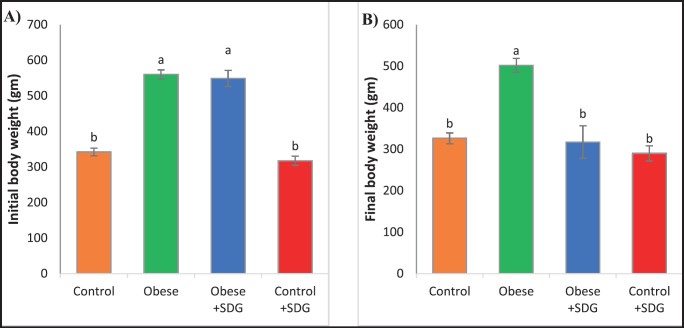
Initial and final body weight (gm) among different groups after 8 and 12 weeks, respectively. (A) The initial body weight. (B) The final body weight at the end of the experiment. The data are articulated as mean ± SD. Statistically significant differences were observed among groups (*p* < 0.05) in the alphabets (a and b) located within the same row.

Another group of embedded paraffin sections was also applied for immunodetection of P53 protein and BCL2 positive cells using Rabbit polyclonal anti-p53 antibody (ab131442) at 1/100 dilution and Anti-Bcl2 Rabbit polyclonal Antibody (ab194583) at 1:100 dilution. All experimental groups’ tissue slices were dewaxed and hydrated. Staining was then conducted in compliance with the producer’s directions and with the 3,3N-diaminobenzidine tertrahydrochloride chromogenic agent (Expose mouse and rabbit-specific HRP/DAB detection kit, abcam; ready-to-use; catalog number: ab80436). There was counterstaining with hematoxylin. For each antigen, three immunolabeled sections per animal (*N* = 10 animals per group) were evaluated.

### Statistical analysis

The information was presented in the form of a range and standard deviation. An analysis of variance was performed to compare the unpaired values of multiple groups. Statistical significance was determined at a level of *p* ≥ 0.05. The analysis was performed using Graph Pad Prism (SAS Institute, Inc., USA), following the methods outlined by Dawson and Trapp [38].

## Results

### Body weight

The initial body weight was taken at the beginning of treatment. The body weight measurements showed a remarkable drop in the weight of the SDG-treated group to the obese group. The results were comparable between the SDG-treated and the control SDG-administrated group with the control group (Fig. 1).

### Biochemical determinations

**Figure 2.**
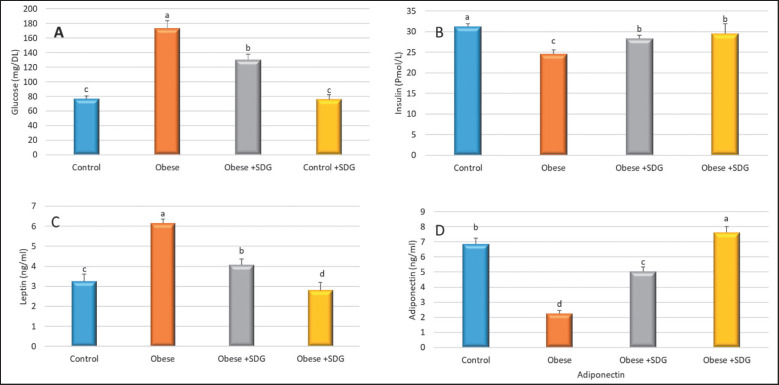
The effect of SDG flax on Biochemical parameters*. *(A) Glucose, (B) INS, (C) leptin, and (D) Adiponectin. The data are expressed as mean ± SD. Statistically significant differences were observed among groups (*p* < 0.05) in the alphabets (a–d) located within the same row.

The group of rats that were fed an HFD exhibited significantly higher levels of serum glucose and leptin in comparison to the control group. This rise, however, was considerably attenuated by the use of SDG extract in the obese group with flaxseed SDG extract. Also, the serum adiponectin and INS levels of the obese group were significantly decreased compared to the control group and the control group with SDG. The obese group treated with SDG significantly increased adiponectin and INS levels (Fig. 2).

**Figure 3.**
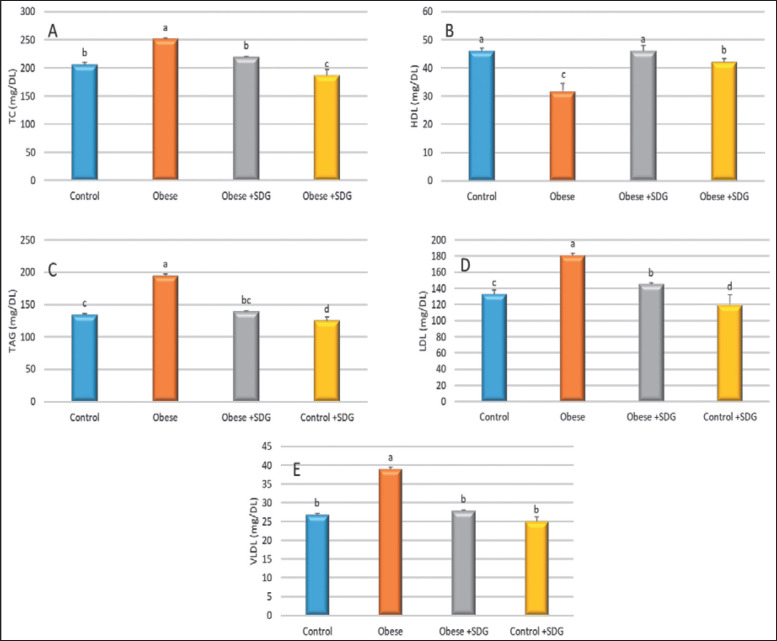
The effect of SDG flax on lipid profile. (A) TC, (B) HDL, (C) TAG, (D) LDL, and (E) VLDL. The data are expressed as mean ± SD. Statistically significant differences were observed among groups (*p* < 0.05) in the alphabets (a and b) located within the same row.

After 12 weeks, serum lipid profiles were taken from all the groups. Similar to the control group, HFD-fed rats had significantly higher concentrations of triglycerides (TAG), total cholesterol (TC), low-density lipoprotein (LDL), and very low-density lipoprotein (VLDL); however, a significant reduction in the lipid status was reported after the administration of SDG. Serum high-density lipoprotein (HDL-C) was considerably higher in the SDG group than in obese rats (Fig. 3).

**Figure 4.**
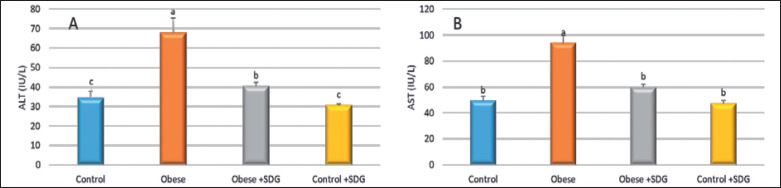
The effect of SDG flax on liver enzymes. (A) ALT and (B) AST. Statistically significant differences were observed among groups (*p* < 0.05) in the alphabets (a and b) located within the same row.

**Figure 5.**
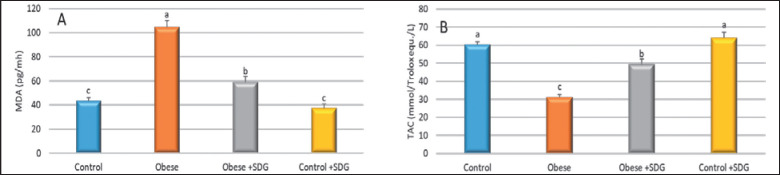
The effect of SDG flax on (A) MDA and (B) TAC. Different alphabets (a–c) within the same row were significantly different among groups *(p < *0.05).

**Figure 6.**
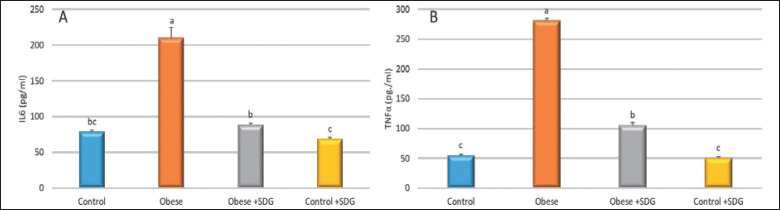
The effect of SDG flax on (A) IL-6 and (B) TNF-α. Data are expressed as mean ± SD. Different alphabets (a–c) within the same row were significantly different (*p < *0.05).

The activities of liver enzymes are presented in Figure 4. The activities of AST and alanine transaminase (ALT) among the obese group were elevated compared to the control and treated groups (*p* = 0.0001). Flaxseed extract supplementation exposed a significant reduction in MDA levels and a significant enhancement of the TAC levels, showing a higher antioxidant activity of SDG flaxseed extract compared to the obese group (Fig. 5). The anti-inflammatory effect was evaluated using the TNF-α and IL-6 rates, where significantly higher levels were found among the obese groups distinguished from the control group. The treated obese group showed a marked decrease in TNF-α and IL-6 concentrations in comparison to the obese group (Fig. 6).

### Molecular findings

**Figure 7.**
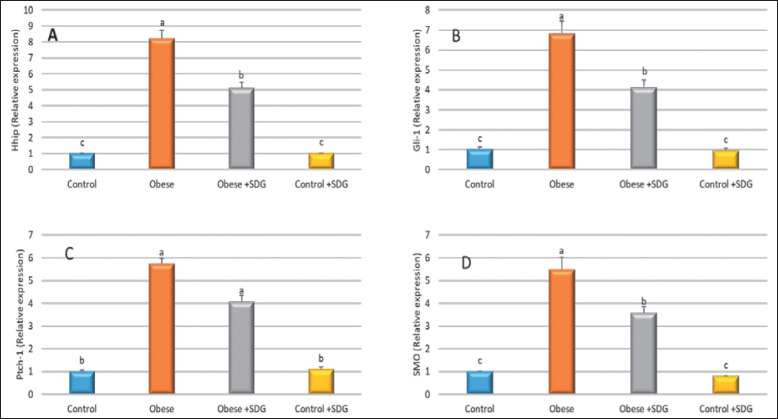
Relative gene expression of Hhog pathway in adipose tissue. (A) Hedgehog interacting protein (Hhip), (B) glioma-associated oncogene (Gli-1), (C) patched (ptch-1), and (D) smoothened (SMO). Data are expressed as mean ± SD. Different alphabets (a–c) within the same row were significantly different among groups *(p < *0.05).

The Hhog pathway in adipose tissue is presented in Figure 7. Obese rats showed significant upregulation in relative mRNA expression of Hhip, glioma-associated oncogene homolog 1 (Gli-1), ptch-1, and smoothened receptor (SMO) compared to the control group. No statistically significant difference was observed between the control group and the group administered with the control SDG. The treated rats showed significant downregulation in the expression of the genes correlated with the obese group.

**Figure 8.**
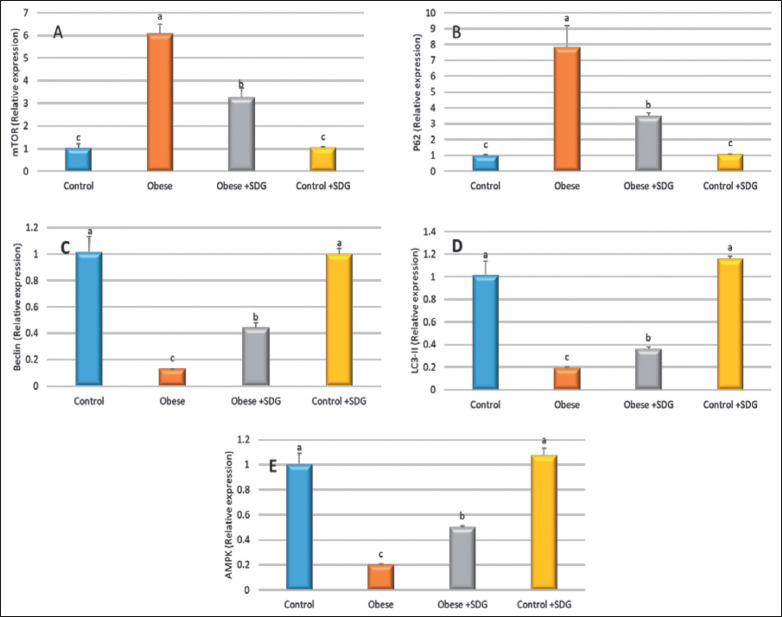
Relative gene expression of autophagy genes. (A) mTOR, (B) ubiquitin-binding protein (P62), (C) Beclin, (D) LC3-II, and (E) AMPK. Data are expressed as mean ± SD. Different alphabets (a–c) within the same row were significantly different among groups *(p < *0.05).

The results presented in Figure 8 demonstrate that there was a notable surge in the relative mRNA expression of mTOR and P62 in rats with obesity as compared to the control group. No statistically significant difference was observed between the control group and the group that received the control SDG administration. The results indicate a noteworthy decline in the expression of the mTOR and P62 genes in the treated rats in comparison to the obese group. The study observed a significant decrease in the relative mRNA expression of Beclin, light chain 3 (LC3)-II, and AMP-activated protein kinase (AMPK) in rats with obesity in contrast to the control group. Compared to the obese group, Beclin, LC3II, and AMPK gene expression exhibited significant upregulation in the treated rats.

### Histopathological investigations

#### H&E staining

### Liver tissue

**Figure 9.**
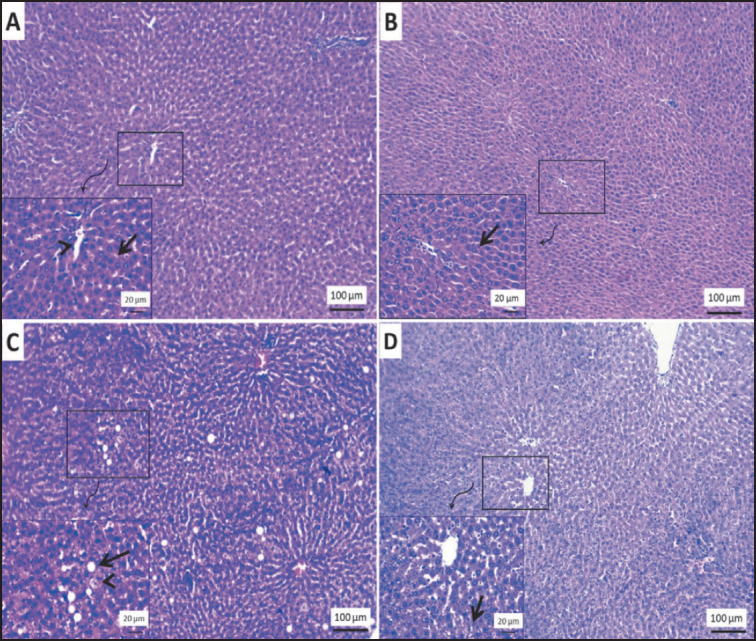
Photomicrograph of H&E stained sections from rat liver showing: (A, B) normal histology of hepatic cords (arrow) and central vein (arrowhead) in the livers of the control group and control-treated rats with SDG flax extract. (C) Variable sizes fat globules, signet ring appeared cell (arrow) and foamy cell (arrowhead) in the obese group. (D) Randomly distributed tiny vacuoles within hepatic cytoplasm (arrow) in the obese group treated with SDG flax extract. The scale bar is 100 μm, and the included figures are 20 μm.

Normal histological structures of hepatic parenchyma were detected in the livers of the control group and control-treated rats with SDG extract (Fig. 9A and B). The liver of the obese group exhibited fatty liver, which was represented by massive areas of variable sizes of fat globules within the cytoplasm of hepatic cells (Fig. 9C). The obese rats treated with flax extract (Fig. 9D) demonstrated a more prominent improvement in the vacuolation of the cytoplasm of the hepatic parenchyma than the obese group.

#### Adipose tissue

**Figure 10.**
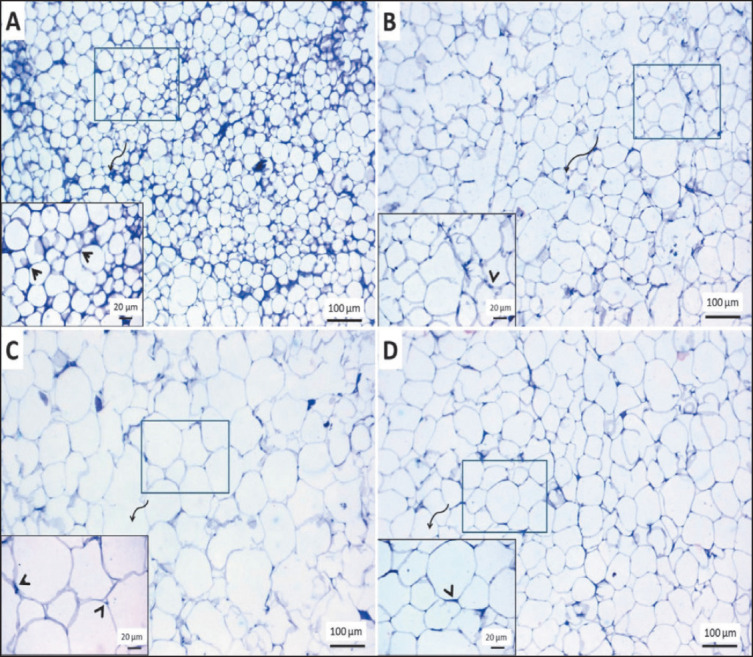
Representative photomicrographs of H&E stained sections from perirenal fat in rats showing: (A, B) Typical histological structures of clear vacuolated fat cells with a compact nucleus at one side (arrowheads) with a minimum amount of extracellular matrix in control and control + SDG groups. (C) Overcrowded large-sized adipocytes with peripherally located nuclei (arrowheads) and a higher number of fatty cysts in the obese group. (D) Uniform vacuolated adipocytes with compressed nuclei (arrowhead) at the periphery and an intact cell membrane in obese group + SDG. The scale bar is 100 μm, and the included figures are of 20 μm.

Normal histological structures of clear vacuolated fat cells with a compact nucleus at one side and a minimum amount of extracellular matrix in the perirenal fat of the control group and flax extract-treated group (Fig. 10A and B). However, the obese group showed large adipocytes with several fatty cysts due to ruptured adipocytes (Fig. 10C). The size of adipocytes in the treated obese group with flax extract (Fig. 10D) appeared to have fewer diameters than the obese group.

### Immunohistochemical results

**Figure 11.**
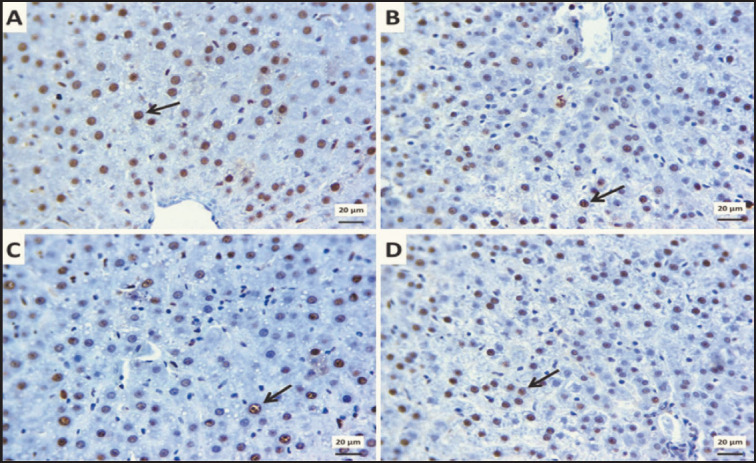
Photomicrographs of immunoexpressed liver sections for P53 showing: Moderate hepatic nuclear expression of P53 (arrows) in the control group (A) and control +SDG group (B). Few immunostained cells of hepatocytes (arrow) among the obese group (C). An intense number of positive staining cells (arrow) in the treated obese group by SDG seeds (D). IHC counterstaining with Mayer’s hematoxylin. The scale bar is 20 μm.

Adipose tissue sections stained against tumor suppressor protein (P53) revealed diffusely distributed nuclear expressions in a moderate number of hepatocytes in the control group (Fig. 11A) and flax seed treated group (Fig. 11B). In contrast, few immunostained cells were seen in the obese group (Fig. 11C). Moreover, the obese group treated with flax seeds showed an intense number of positive staining cells (Fig. 11D).

**Figure 12.**
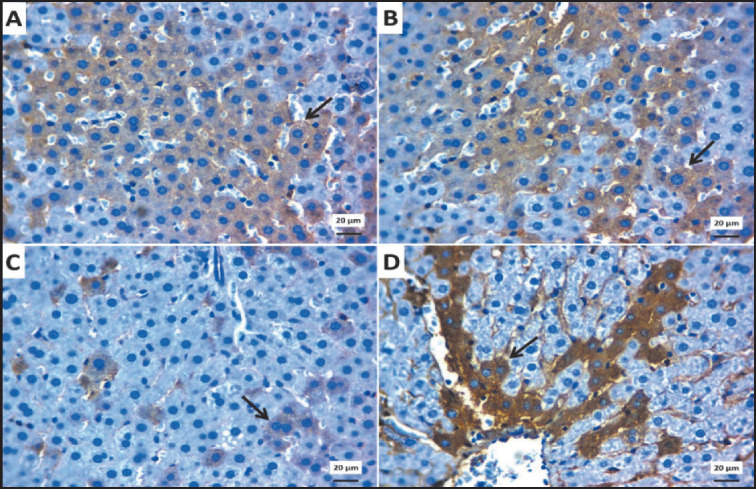
Photomicrographs of immunoreactivity for Bcl2 in the liver showing: Increased immunostaining cells within hepatocytes (arrow) for Bcl2 in control (A) and control group treated with SDG flax (B). Few expressed cells (arrow) for BCl2 in the Obese group (C). Moderate number of immunoexpressed cells (arrow) for Bcl2 in the obese group treated with SDG flax (D). IHC counterstaining with Mayer’s hematoxylin. The scale bar is 20 μm.

The control group (Fig. 12A) and flax seed groups (Fig. 12B) for Bcl2 showed markedly increased immunoreactive cells. However, the treated obese group with flax seeds showed more expressed cells (Fig. 12D) than the obese group (Fig. 12C).

## Discussion

Flaxseed extract, a natural plant-based product derived from the seeds of the *Linum usitatissimum* plant, has been increasingly studied for its positive effects on human health. In particular, the impact of flaxseed extract on glucose, INS, adiponectin, and leptin levels has been the focus of recent research. These biomarkers are essential indicators of metabolic health and are associated with the development of chronic conditions such as diabetes, obesity, and cardiac and vascular diseases. Thus, understanding the impact of flaxseed extract on these biomarkers may help improve the health of individuals at risk for these conditions [39].

The body weight of the treated obese group showed a considerable reduction compared to the obese group. The levels of glucose and leptin showed a significant attenuation among the SDG extract-treated obese group compared to the obese group. Also, serum INS and adiponectin levels in the obese group were significantly inhibited compared to all the groups, with a significant rise among the obese group treated with SDG. The extract has also shown beneficial effects on the lipid profile of the treated group compared to the obese group. Similarly, flaxseed’s water-soluble fibers and alpha-linolenic acid account for the weight loss noticed in treated groups after supplementation with the seed. The seed is thought to play a role in this weight loss by dissolving harmful dietary fats in the digestive tract so that they can be flushed out of the body rather than being stored. Increased sensitivity to the leptin hormone is one-way alpha-linolenic acid that controls weight loss [40]. This acid also stimulates thyroid function, speeding up metabolism and boosting the thermogenic process in brown adipose tissue [41,42].

Flaxseed’s rich lignan content and potential antioxidant effectiveness have been linked to its hypoglycemic impact by increasing the sensitivity of peripheral INS and decreasing gluconeogenesis and glycogenolysis [8]. In addition, glucose transport, INS secretion, action, and correction of hyperglycemia connected to INS resistance in Polycystic ovary rats are all improved by dietary flaxseed [43].

According to the present findings, a mixture of flaxseed and barley decreased glucose, cholesterol, LDL, and triacylglycerols and increased HDL [44]. Previously, Elshal et al. [45] showed that flaxseed was effective in lowering cholesterol, glucose, TAG, and LDL while increasing HDL compared to diabetic rats. Additionally, a blend of flaxseed and pumpkin seed resulted in a 47% reduction in blood cholesterol and triacylglycerols compared to diabetic rats, as the mixture provided phytosterols, polyunsaturated fatty acids, tocopherols, and carotene [46]. Furthermore, flaxseed supplementation decreased cholesterol and increased serum HDL in streptozotocin-induced diabetic hamsters, unlike the control diabetic hamster group [47].

Moreover, Khalesi et al. [48] examined the results of different raw and heated flaxseed concentrations on the lipid profile of normal diet-fed rats. The group fed 30% flaxseed had the most significant decline in cholesterol, LDL, and triacylglycerols and a tremendous rise in HDL, followed by the groups provided with lower flax concentrations. Another study with comparable results linked these benefits to flaxseed’s soluble fiber and SDG content [49].

Flaxseed extract supplementation showed a notable decrease in AST, ALT, MDA, IL-6, and TNF-α levels, with a significant increase in TAC levels, showing a higher antioxidant activity and anti-inflammatory activity of SDG flaxseed extract compared to the obese group.

Similarly, MDA, a blood diagnostic tool of lipid peroxidation, was decreased by Flax diets. However, the preventative strategy was more inhibitive than the therapeutic approach with diclofenac. Serum TAC was used to assess the antioxidant capacity as the FLAX treatments raised TAC, with the impact being more pronounced in the preventative plan than in the therapeutic diclofenac group [50]. Dietary lignans controlling the inflammatory response suppressed pro-inflammatory cytokines (TNF-α, IL-6) [51,52].

Hedgehog (Hh) signaling is correlated with the regulation of adipose tissue. To illustrate, Hh signaling suppresses adipocyte development *in vivo* and *in vitro* [53,54]. Hh is a critical component in adipose tissue progression in animals since fat-specific Hh stimulation in mice resulted in a complete decrease in white (WAT) but not brown (BAT) adipose tissue. Consequently, WAT and BAT adipogenesis are significantly controlled by Hh [54]. Moreover, Hh signaling changes the cellular metabolism through a different, non-canonical Hh signaling route toward a Warburg-like glycolytic state [55].

This study is intended to examine the modulation of Hh ligands and their signaling components in obesity. Using SDG flaxseed extract, the relative mRNA expression of Hhip, Gli-1, ptch-1, and SMO in obese rats increased significantly in contrast to the control group. Contrary to the obese group, the expression of the genes was significantly negatively regulated in the treated rats.

The study found that Shh, Ihh, and Hhip were upregulated in the groups fed saturated and unsaturated fatty diets. However, it was observed that Ihh was significantly downregulated in the HFD-fed group compared to the control group. The expression of Ptch1 was elevated considerably in the HFD-2 group, while the expression of SMO showed a similar increase in both experimental groups compared to the control group. The initiation of the Hh signaling pathway is triggered by the binding event between Hh and the receptor, which results in the derepression of SMO. The triggering of hepatic stellate cells, induction of liver fibrosis, and upregulation of numerous fibrogenic genes have been observed due to elevated hepatic expression of Shh [56–58]. Also, Gli-1 was elevated in the HFD-1 group, but in the D-2 group, expression among those genes followed the same pattern as in the control group [56]. It has been observed that elevated Gli-1 levels serve as a marker for hepatocellular carcinoma [59].

According to our knowledge and a review of the available studies, no information was encountered regarding the effect of flaxseed extract on the Sonic Hedgehog pathway (Hh) in obese rats, from which it could be concluded that using SDG flax extract could alleviate obesity by interfering with the activation of the Hh pathway.

The current research additionally assessed the impact of the plant extracts on obese rats via the modification of autophagy. Compared to the control group, the relative mRNA expression of mTOR and P62 was significantly upregulated in obese rats. No significant change was discovered between the control group and control + flax. Compared to the obese group, the treated rats exhibited considerable downregulation of the mTOR and P62 genes.

In addition, relative mRNA expression of Beclin, LC3-II, and AMPK was significantly reduced in obese rats as compared to the control group. Compared to the obese group, the treated rats exhibited considerable upregulation of Beclin, LC3-II, and AMPK genes.

Autophagy is an evolutionarily catabolic process in lysosomes in which damaged organelles and proteins are destroyed and used to produce new organelles and adenosine triphosphate [60].

Aberrant alterations, either an increase or a reduction in autophagy, are the primary etiology of several human illnesses, such as cancer, cardiovascular disorders, diabetes, obesity, and aging. Specifically, obese humans and experimental models of diet-induced obesity or genetically susceptible individuals have been shown to have altered autophagy (increased or decreased) [61–63].

Evaluation of aberrant autophagy alterations related to obesity may be somewhat complex since it depends on the nature, length, obesity models, the evaluated cells or tissues, and the employed autophagy analysis tools [64].

In addition, obesity is typically accompanied by abnormalities in autophagy. On the contrary, laboratory animals exhibit changes in lipid metabolic activities, liver steatosis, and the manifestation of obesity or type 2 diabetes mellitus phenotypes when specific autophagy protein sequences or autophagy-controlling molecules are deleted or mutated, either globally or in a tissue-specific manner [65], indicating a crucial part of autophagy in the initiation and progression of obesity and its related problems. The scientific community has widely acknowledged that alterations in crucial metabolic indicators, including glucose, amino acids, and lipids, can function as nutritional tools to regulate autophagy. Consequently, these changes can impact cellular metabolism, INS sensitivity, adipogenesis, cell viability, and organ performance [66,67].

According to the recently published studies, there was a lack of studies assessing the effects of lignans on obese rats through the modulation of autophagy. However, previous studies evaluated the effects of lignans on the modulation of autophagy in many diseases, including obesity [68–70]. Lignans are a vital class of polyphenols that cause cancer cell growth limitation and death via apoptosis and autophagy with low harm to non-transformed cells. Lignan consumption reduces the risk of many malignancies by targeting many signaling molecules and pathways [71–74]. These natural chemicals may suppress carcinogenesis, tumor development, and metastasis. There is accumulating evidence that natural lignans, including magnolol and honokiol, offer significant anticancer effects against a variety of human cancer types [75].

Accordingly, Lignan vitexin 6 (VB6) was discovered to activate autophagy and programed cell death in different cancer cells. Time- and concentration-dependent relationships were observed between VB6-induced apoptosis, Bax activation, cleaved caspase-3, poly (ADP-ribose) polymerase, and Bcl-2 decreased expression. Beclin-1 and LC3-II, which are indicators of autophagy, arise progressively after VB6 therapy. Following VB6 therapy, Jun N-terminal kinase phosphorylation increased, along with P-C-Jun and P-Bcl-2 expression [76].

Moreover, lignans block downstream mTOR effector molecules. The suppression of the mTOR pathway increases autophagy directly since mTOR inhibits autophagy through Ulk1 [77] and reduces the translation of proteins, cellular growth, and proliferation, impacting cancer. Consequently, lignans block the mTOR pathway, resulting in an anti-colitis effect in immune cells, stress of the endoplasmic reticulum, death of liver cancer cells, and β-amyloid clearance in Alzheimer’s disease [68–70].

In addition, lignans have been found to suppress mTORC1 activation in Th17 cells through binding to ER [71]. In human ER-positive MCF-7 breast cancer cells, lignans inhibited mTOR downstream effector molecules, causing autophagy-induced cell death with downregulation of ER [72].

In the same respect, trachelogenin lignans continuously promoted cytoplasmic vacuolization, cell death, and the production of autophagosomes mediated by boosting LC3 activation and modifying Beclin-1 expression levels [73]. Different studies have shown that polyphenols, abundant in plant-based diets and drinks, may effectively control autophagy in several forms of cancer [74].

As for the lignan’s effect on Bcl-2 and P53 genes responsible for apoptosis modification, the treated groups showed significant up-regulation of the genes in adipose tissue and liver tissue. Repair and apoptosis may be triggered by p53, which pauses the cell cycle, scans for potential DNA damage, and, if required, promotes repair or initiates cellular death [78]. It is possible that apoptotic abnormalities, such as p53 deletion, permit an aberrant rise in adipocyte size [79]. Interestingly, the pro-survival protein BCL-2 activation in adipocytes correlates adversely with body mass index [80]. Nevertheless, the processes that govern adipocyte death and growth remain poorly known. The decrease in BCL2 gene expression when BMI rises lends credence to the theory of a change in equilibrium. Regarding this topic, the verified truth is that in obesity, free fatty acids produced from adipose tissue stimulate the death of pancreatic cells by an endoplasmic stress response and suppress the expression of the antiapoptotic protein BCL2 [80]. Consequently, there were concentration- and time-dependent connections between VB6-lignan-induced apoptosis, Bax activation, cleaved caspase-3, poly (ADP-ribose) polymerase, and Bcl-2 reduced expression.

Following VB6 treatment, P-Bcl-2 expression increases [76]. In addition, prior research has shown that flaxseed administration dramatically elevates p53 mRNA in MCF-7 and MDA-MB-231 cells, ultimately causing apoptosis [81].

## Conclusion

The study demonstrated the role of SDG flaxseed lignan-enriched extract as an anti-inflammatory, antioxidant, and anti-obesity agent. It modulates obesity markers such as glucose, INS, leptin, adiponectin, lipid profile, and liver enzymes. Also, its effect on regulating the Hhog pathway, which is implicated in adipose tissue regulation, is another process for protecting against obesity. Regarding the autophagy and apoptotic effects of the extract, it showed a significant stimulation or obstruction of autophagy initiation and apoptosis depending on the regulation of autophagy and apoptotic-related genes. SDG flaxseed lignan-enriched extract has demonstrated anti-inflammatory, antioxidant, autophagy, apoptotic, and anti-obesity effects among obese rats. Thus, it may be used after advanced future research to prevent and treat obesity and its related complications by modulating oxidation, inflammation, autophagy, and apoptosis.

## List of Abbreviations

ATP: Adenosine Triphosphate, AMPK: AMP-activated protein kinase, DAB: 3,3N-Diaminobenzidine Tertrahydrochloride, ERs: Estrogen receptors, Gli-1: Glioma-associated oncogene homolog 1, Hh: Hedgehog, Hhip: Hh interacting protein, HRP: horseradish peroxidase, IL-6: Interleukin-6, LC3: Light chain 3, MDA: Malondialdehyde, mTOR: Mammalian target of rapamycin, P62: Ubiquitin-binding protein, PCO: polycystic ovary, ptch-1: Protein patched homolog 1, SDG: Secoisolariciresinol diglucoside, SMO: Smoothened receptor, TAC: Total antioxidant capacity, TNF-alpha: Tumor necrosis factor-alpha, VAT: Visceral adipose tissue.
